# HTLV-1/-2 and HIV-1 co-infections: retroviral interference on host immune status

**DOI:** 10.3389/fmicb.2013.00372

**Published:** 2013-12-23

**Authors:** Elisabetta Pilotti, Maria V. Bianchi, Andrea De Maria, Federica Bozzano, Maria G. Romanelli, Umberto Bertazzoni, Claudio Casoli

**Affiliations:** ^1^GEMIB Laboratory, Center for Medical Research and Molecular DiagnosticsParma, Italy; ^2^Department of Health Sciences, University of GenovaGenova, Italy; ^3^Center of Excellence for Biomedical Research, University of GenovaGenova, Italy; ^4^IRCCS AOU San Martino-IST GenovaGenova, Italy; ^5^Department of Life and Reproduction Sciences, University of VeronaVerona, Italy

**Keywords:** HTLV, HIV-1, co-infection, cytokines, chemokines, JAK/STAT, miRNA, natural killer cells

## Abstract

The human retroviruses HIV-1 and HTLV-1/HTLV-2 share similar routes of transmission but cause significantly different diseases. In this review we have outlined the immune mediated mechanisms by which HTLVs affect HIV-1 disease in co-infected hosts. During co-infection with HIV-1, HTLV-2 modulates the cellular microenvironment favoring its own viability and inhibiting HIV-1 progression. This is achieved when the HTLV-2 proviral load is higher than that of HIV-1, and thanks to the ability of HTLV-2 to: (i) up-regulate viral suppressive CCL3L1 chemokine expression; (ii) overcome HIV-1 capacity to activate the JAK/STAT pathway; (iii) reduce the activation of T and NK cells; (iv) modulate the host miRNA profiles. These alterations of immune functions have been mainly attributed to the effects of the HTLV-2 regulatory protein Tax and suggest that HTLV-2 exerts a protective role against HIV-1 infection. Contrary to HIV-1/HTLV-2, the effect of HIV-1/HTLV-1 co-infection on immunological and pathological conditions is still controversial. There is evidence that indicates a worsening of HIV-1 infection, while other evidence does not show clinically relevant effects in HIV-positive people. Possible differences on innate immune mechanisms and a particularly impact on NK cells are becoming evident. The differences between the two HIV-1/HTLV-1 and HIV-1/HTLV-2 co-infections are highlighted and further discussed.

## INTRODUCTION

Microbes that infect the same host may positively influence each other’s replication, or fight for supremacy (Margolis, 2003; Kannangara et al., 2005). In recent years, different data have demonstrated that a microbe, in order to favor its own replication, can attenuate or support the infection of other infecting agents by altering the host immune system (Lisco et al., 2009). The role of human T lymphotropic viruses type 1 and type 2 (HTLV-1 and HTLV-2) as determinants of HIV-1 disease during co-infection has been widely studied, but is still a matter of speculation.

Since HTLV-1, HTLV-2, and HIV-1 have common modes of transmission, it is not surprising that co-infection is a frequent condition especially among people with high risk behaviors, as needle sharing and unprotected sexual contact. Though human retroviruses have worldwide distribution (Goubau et al., 1992; Vrielink and Reesink, 2004; Proietti et al., 2005), dually infected subjects have been mainly diagnosed in large metropolitan area or in endemic regions (Briggs et al., 1995; Dezzutti et al., 1998; Araujo et al., 2002; Morimoto et al., 2005; Magri et al., 2012, 2013). Because HTLV screening is not routinely performed in many countries and is not always recommended by physicians to outpatients, the seroprevalence of co-infection is underestimated (Beilke, 2012; Pinto et al., 2012).

HTLV-1, HTLV-2, and HIV-1 share similar genomic organization and tropism for immune cells, in particular CD4^+^ and CD8^+^ T cells. However, the finality of their viral cycle is different. In infected patients, HIV-1 is generally present as a virion (either cell-associated or cell-free) and the provirus is detected clearly in a minority of cells (Josefsson et al., 2011), whereas HTLVs are prevalently integrated in their target cells and the propagation of infection occurs by clonal expansion of infected cells (Bangham, 2003).

To understand whether HTLVs may accelerate or attenuate HIV-1 progression, several studies have interpreted HIV-1-associated clinical outcomes, taking into account laboratory records as CD4 mean cell count and HIV-1 viral load. However, conflicting data have emerged on both HTLV-1 and HTLV-2 interactions with HIV-1 and the debate is still open. The lack of uniformity of used criteria may explain this discrepancy, including differences in sampling, variations in outcome surveillance, with other parameters, such as HTLVs and HIV-1 seroconversion time and sex, not always defined. Furthermore, some authors believe that matching patients by immune markers is not a good strategy (Beilke et al., 2004). When not well-defined, also anti-HIV-1 therapy, considering its effect on immunologic host factors and potentially on replication of co-invading agents, could make the comparison of data coming from different studies difficult.

Concerning HTLV-1/HIV-1 co-infection, a more rapid HIV-1 disease progression and a lower mean survival time in co-infected individuals compared to HIV-1-mono-infected patients (Brites et al., 2001) was seen. Additionally, an increased risk to develop tropical spastic paraparesis/HTLV associated myelopathy (TSP/HAM) and other neurodegenerative conditions was found in dually infected subjects (Tulius Silva et al., 2009). Also, a recent work reported a higher mortality and shortened survival rate in HTLV-1/HIV-1-co-infected children compared to HIV-1-mono-infected patients irrespective of baseline CD4 cell count (Pedroso et al., 2011).

On the contrary, other researchers observed a delayed HIV-1 disease progression in HTLV-1-co-infected patients when compared to HIV-1-mono-infected individuals (Shibata et al., 1989; Page et al., 1990; Chavance et al., 1995; Beilke et al., 2004). In according to these findings, a higher survival rate was reported for HIV-1/HTLV-1-co-infected subjects (Brites et al., 2001, 2005).

It is noteworthy that the impact of HIV-1 on HTLV-1 replication was also investigated. Seroepidemiologic studies showed that HIV-1 positive subjects are more susceptible to HTLV-1 infection, and vice versa (Harrison et al., 1997). Moreover, the HIV-1 Rev protein was found to enhance HTLV-1 gene expression, by interacting with 5’-RU5 region of the HTLV-1 genome (Kubota et al., 1998). A later study, in which the quantification of HTLV-1 and HIV-1 DNA load was determined, suggested that HIV-1 co-infection does not affect HTLV-1 proviral load in peripheral blood compartments (Cesaire et al., 2001).

Similarly to HTLV-1/HIV-1, contrasting results were also reported for HTLV-2/HIV-1 co-infection. However, it is now generally accepted that HTLV-2 exerts a negative effect on HIV-1 replication. In fact, several authors have associated HTLV-2 co-infection with a better outcome for HIV-1 positive individuals (Beilke et al., 2004; Casoli et al., 2007). Earlier studies did not observe this effect and ascribed the lack of significant associations between co-infection and progression to AIDS or death to the absence of clear evidence of HTLV-2 as pathogenic agent (Visconti et al., 1993; Hershow et al., 1996).

HTLVs interfere with HIV infection by mechanisms that appear to be complex and multilayered. More specifically, HTLVs can act on HIV-1 expression directly at molecular levels or indirectly by modulating the expression of immune host factors. This in turn can be induced by HTLVs directly or through cellular activation.

Thus, the intimate relationship between the HIV-1 life cycle and the activation state of cells supporting viral replication results in a dynamic interaction between co-infecting agents and HIV-1 replication in dually infected individuals.

HTLV-1 differs from HTLV-2 in regulating cellular activation of target cells. More specifically, HTLV-1-infected subjects present a prevalence of highly activated cells, while HTLV-2-infected individuals hold up a lower cellular activation status (Nagai et al., 2001; Bovolenta et al., 2002a; Goon et al., 2004). These divergent conditions may contribute to explain the different impact of the two types of HTLV on HIV-1 infection. In particular, for HTLV-2 it was observed that HIV-1/HTLV-2-co-infected patients showed a reduced HIV-1 replication presumably due to lower levels of T cell activation (Bassani et al., 2007).

In this review, we report and discuss recently published data on host immunomodulating factors involved in retroviral interference, and in that regard we also point at other cellular and molecular components that may be considered potentially good candidates.

## CYTOKINES/CHEMOKINES

The role of the cytokine/chemokine network as strategic weapon in germ warfare has been extensively discussed (Margolis, 2003). Infecting agents benefit from their ability to drive immune reactions mediated by cytokines and chemokines. This favorable condition for a microbe could be adverse or advantageous for a co-pathogen.

Thus, the perturbation of the immune system, including host cytokine synthesis, induced by HTLV-1 and HTLV-2 infections (Hollsberg, 1999) could have positive or negative impacts on HIV-1 replication. Such immune activation is mainly modulated by HTLV-1 and HTLV-2 Tax proteins (Tax-1 and Tax-2).

Regarding HTLV-1/HIV-1 co-infection, it was reported that Tax-1 up-regulates HIV-1 expression (Bohnlein et al., 1989) by activating the transcriptional factor NF-κB that recognizes two binding sites in the U3 region of the HIV-1 LTR (Leung and Nabel, 1988). In addition, Tax-1 enhances the expression of several cellular proteins, including transcription factors and cytokines as IL-2, tumor necrosis factor α (TNF-α), and others (Banerjee et al., 2007; Boxus et al., 2008). More specifically, some of these cytokines as TNF-α or IL-1β are capable of triggering HIV-1 transcription through a NF-κB-dependent mechanism (Siebenlist et al., 1994). Tax-1 is also responsible for the induction of cytokine receptor expression (Franchini and Streicher, 1995). This state of activation was frequently observed among co-infected subjects who showed a sharp drop in CD4 cell count and rapid progression of HIV-1 disease (Bartholomew et al., 1987; Page et al., 1990; Pagliuca et al., 1990; Gotuzzo et al., 1992; Schechter et al., 1994; Fantry et al., 1995).

However, other studies describe detrimental effects on HIV-1 infections (Harrison et al., 1997; Beilke et al., 2004, 2007). Recently, Abrahao et al. (2012) observed a higher production of IL-2 and IFN-γ in HIV-1/HTLV-1-co-infected individuals than in HIV-1 or HTLV-1-mono-infected individuals. Moreover, while IL-6 and IL-10 levels were similar in all infected groups, IL-4 production was lower in HTLV-1-mono-infected individuals. These findings support the notion that high levels of Th-1 cytokines in co-infected patients provide adverse conditions for HIV-1 infection, suggesting a predominant role of HTLV-1 over HIV-1. This hypothesis is supported by the evidence that IL-2 suppresses HIV-1 replication in some HTLV-1-infected cell lines by inducing APOBEC3G expression (Oguariri et al., 2013).

For what concerns HTLV-2, it was reported that PBMCs derived from HTLV-2 seropositive individuals undergo spontaneous proliferation in short-term cultures in association with the secretion of several cytokines, including TNF-α, IL-5, IL-6, and IFN-γ (Dezzutti et al., 1998).

By studying the cytokine pattern in HTLV-2/HIV-1-co-infected subjects, we determined that HTLV-2 drives immune activation to implement the secretion of cytokines as GM-CSF and IFN-γ (Pilotti et al., 2007), which are capable to induce a “protective” Th1 response against HIV-1 (Creery et al., 2004), since a dominant Th2 profile seems to favor HIV-1 progression (O’Garra, 1998).

Other important immune correlates able to facilitate or suppress HIV-1 infection are the chemokines and their cellular receptors. *In vivo*, HIV-1 prevalently targets immune cells expressing the surface receptor CD4 that mediates virus binding and membrane fusion together with chemokine co-receptors (CCR5 and CXCR4; Kowalski et al., 1987; Lasky et al., 1987). Thus, changes in conformation status or surface availability of these molecules may, in turn, modify HIV-1 disease progression. Beside genetic modifications, the co-receptors expression is mainly affected by the binding of natural ligands. In particular three CCR5 binding chemokines, CCL3, CCL4, and CCL5, act as major HIV-1-suppressive factors.

Because three chemokines are released by both cultured T cells and primary CD8^+^ T cells in response to HTLV infection (Scarlatti et al., 1997), it was supposed that their up-regulation could explain HIV-1 inhibition observed during co-infection. The fact that HTLV-1-specific CD8^+^ cytotoxic T lymphocyte (CTL) clones derived from patients with HAM/TSP are actively producing CCL3 and CCL4 chemokines (Cocchi et al., 1995), reinforces the hypothesis that HTLV-1 can influence HIV-1 replication via chemokine expression and release.

As HTLV-2-infected cells become activated, they spontaneously proliferate and produce high levels of various cytokines and chemokines (Casoli et al., 1998, 2000; Lewis et al., 2000; Bovolenta et al., 2002b; Bassani et al., 2007). We observed that up-regulation of CCR5-binding chemokine expression occurs in cultured PBMCs from HTLV-2/HIV-1-co-infected individuals in comparison to HIV-1-single-infected individuals. In particular, we demonstrated that CCL3 secretion is responsible for anti-HIV-1 activity in PBMC cultures from co-infected subjects (Casoli et al., 2000). Lewis et al. (2000) associated the spontaneous synthesis of CCR5 binding chemokines to the ability of HTLV-2 regulatory proteins to transactivate *CCL4* and *CCL5* gene promoters. Also, we found that an isoform of CCL3, namely CCL3L1, which is considered the most potent anti-R5 HIV-1 chemokine (Visconti et al., 1993), was preferentially induced by HTLV-2 (Pilotti et al., 2007). Up-regulation of this chemokine leads to CCR5 down-modulation and subsequent receptor internalization (Townson et al., 2002). Although other groups have shown that the HIV-1 susceptibility is associated with *CCL3L1* gene dose, which is variable among individuals, we demonstrated that HIV-1 inhibition occurred in HTLV-2-co-infected subjects was independent by *CCL3L1* copy number, and that enhanced *CCL3L1* expression was presumably stimulated by Tax-2 protein at the transcriptional level (Pilotti et al., 2007). High levels of GM-CSF and IFN-γ secreted by PBMCs from HTLV-2-infected individuals were found to contribute to HIV-1 interference via CCR5 down-modulation (Pilotti et al., 2007).

Two recent works confirmed the pivotal role of Tax proteins in inducing CC-chemokine synthesis. The first paper reported that both recombinant Tax-1 and Tax-2 induce high levels of CC-chemokines which in turn cause CCR5 down-regulation in cultured PBMCs (Barrios et al., 2011), and the second article demonstrated that Tax-2 transactivates CC-chemokines production in cultured monocyte-derived macrophages (Balistrieri et al., 2013).

## JAK/STAT SIGNALING

HTLV-1 and HTLV-2 can efficiently transform human T cells *in vitro* but significantly differ in pathogenicity. The Janus kinase (JAK)/signal transducer/activator of transcription (STAT) signaling pathway (JAK/STAT) is constitutively activated in HTLV-1-transformed cells. This may occur by autocrine stimulation of IL-2, IL-9, and IL-15 cytokines, and IL-2 and IL-15 receptor expression, as a result of Tax-induced NF-κB expression, which in turn stimulate lymphocyte proliferation (Migone et al., 1995; Mariner et al., 2001; Chen et al., 2008). HTLV-1 Tax protein is crucial for viral replication and for initiating malignant transformation and is able to inhibit host antiviral signaling via NF-κB-dependent induction of suppressor of cytokine signaling protein 1 (SOCS1) to evade innate immunity (Charoenthongtrakul et al., 2011). In T cells transformed *in vitro* by HTLV-1, the JAK/STAT activation correlates with the transition from an IL-2 dependent to an IL-2 independent phase of growth (Migone et al., 1995; Xu et al., 1995). In contrast to HTLV-1, the activation status of the JAK/STAT pathway is not constitutively activated in HTLV-2-transformed T cells. However, this pathway could be induced upon IL-2 treatment of the cells. Similarly, the constitutive activation of STAT1, STAT3, and STAT5, as well as the phosphorylation status of JAK kinases (JAK3 and JAK1), observed in HTLV-1-transformed T cell lines, was not detected in HTLV-2-transformed T cells (Mulloy et al., 1998). However, we showed that the ability of human CD34^+^ IL-3 dependent TF-1 cell line to proliferate after HTLV-2 exposure in conditions of IL-3 deprivation is following the production of the GM-CSF and IFN-γ, through the activation of the JAK/STAT pathway (Bovolenta et al., 2002b). Previously, it was demonstrated that a signature of PBMC freshly derived from HIV-1 infected individuals represents the constitutive activation of a C-terminal truncated STAT5 (STAT5Δ) and STAT1 (Bovolenta et al., 1999).

When analyzing the levels of STATs in HTLV-2 mono-infected and HTLV-2/HIV-1-dually-infected individuals, we observed that these factors are not activated in PBMCs of HTLV-2-mono-infected unless they are cultured *in vitro*, in the absence of any mitogenic stimuli, for at least 8 h (Bovolenta et al., 2002a). The emergence of STAT activation, mainly of STAT1, appears to be related to the secretion of IFN-γ. Of note, this is a characteristic feature of both HTLV-2 and HIV-1-mono-infected individuals. Surprisingly, HTLV-2/HIV-1 co-infection resulted in a low/absent STAT activation *in vivo*, thus correlating with a diminished secretion of IFN-γ in *ex vivo* cultivated PBMCs (Bovolenta et al., 2002a). These findings indicate that HTLV-2 and HIV-1 infection can prime T lymphocytes for STAT1 activation, but they also highlight that an interference is exerted by HTLV-2 on HIV-1-induced STAT1 activation. These results clearly suggest that HTLV-2 may interfere with HIV-1 infection at multiple levels. The observation that PBMCs obtained from both HIV-1- and HTLV-2-infected individuals activate STAT1 as a consequence of the spontaneous release of IFN-γ is supported by previous findings indicating an up-regulation of this cytokine following either HIV-1 or HTLV-2 infection (Dezzutti et al., 1998; Levine et al., 2002). An enhanced transcription of IFN-γ is also induced by the HTLV-2 Tax trans-activator (Nimer et al., 1989; Brown et al., 1991). A large body of evidence points to an increased level of either IFN-γ or its correlates (such as neopterin or IP-10) in the plasma/serum of HIV-1-infected individuals (Poli et al., 1994), explaining the low but detectable constitutive STAT1 activation observed in HIV-1-infected. This correlation was not seen in HTLV-2-infected individuals (Bovolenta et al., 1999). Therefore, T cells from both HIV-1- and HTLV-2-infected individuals share a constitutive priming for IFN-γ secretion and, consequently, for STAT1 activation; in contrast, only HIV-1 infection is characterized by activation of STAT5Δ *in vivo* (Bovolenta et al., 1999). Because these factors are absent in both HTLV-2 mono-infected and HTLV-2/HIV-1 co-infected individuals, this would reflect the higher pathogenic potential of HIV-1 with respect to HTLV-2, but also highlights a dominant position of HTLV-2 over HIV-1 in terms of maintaining T cells in a primed but not completely STAT5Δ activated state. Since there is evidence that HTLV-2/HIV-1 co-infection is frequently associated with a state of long-term non-progression (LTNP) of HIV-1 disease (Giacomo et al., 1995; Magnani et al., 1995), HTLV-2 infection, and co-infection with HIV-1, represent an important model to better understand the interaction between human exogenous retroviruses and the immune system. IFN-γ-related priming for STAT1 activation may be an alarming signal that biases the immune response toward a Th1-model of containment of HTLV-2 infection, overcome by a peculiar STAT5Δ activation in HIV-1-infected individuals. Concerning the role of JAK/STAT in HTLV-1/HIV-1 co-infection no results have yet been reported.

## NATURAL KILLER CELL ACTIVITY

Evidence has been accumulating on the specific targeting of innate immune defenses, and in particular of natural killer (NK) cells, by chronically replicating viruses (Marras et al., 2011). Successful weakening of NK cell response represents a critical step for virus persistence, since NK cells are involved in patrolling peripheral tissues for immediate defense against virus or tumor aggression as well as in cross-talking with critical components of innate immunity, including monocytes and dendritic cells, leading to relevant downstream impact on the shaping of adaptive immune responses (Vivier et al., 2011).

Natural killer cells main cytolytic and cytokine productive function is tightly controlled by a wide array of activating NK cell receptors which alone, or in combination with Toll-like- o cytokine- receptors, are responsible for triggering the NK cell functional program (Vivier et al., 2011). Inhibitory NK cells receptors (i.e., KIRs, NKG2A/CD94, CD85j, IRP60, SIGLEC-7), which are mainly, but not exclusively, HLA class I-specific, provide a negative regulatory signal that is able to override any triggering receptor signaling in the presence of the appropriate cognate receptor. Thus, proper NK cell function may occur only in the presence of activating receptor triggering, with reduced or absent overriding control by inhibirtory receptors sensing the respective ligands on target cells. For example, in the absence of MHC class I expression induced by virus down-modulation, the induction in the infected cells of ligands (e.g., MIC-A, MIC-B, ULBPs, Nectin-2, PVR, etc.) recognized by activating NK cell receptors would result in NK cell activation, cytokine/chemokine production, and cytotoxic activity. Several viruses and mycobacteria exploit altered expression of natural/cytotoxicity receptors in NK cells such as HIV-1 (De Maria et al., 2003), influenza (Arnon et al., 2001; Mandelboim et al., 2001; Gazit et al., 2006), HCV (Bozzano et al., 2011), mycobacterium tuberculosis, and Calmette–Guerin (Vankayalapati et al., 2002, 2004; Bozzano et al., 2009; Marras et al., 2012; reviewed in Bozzano et al., 2012 and in Marras et al., 2011), or skew the NK cell peripheral repertoire inducing expansion of NKG2C^+^ NK cell subsets as is the case for CMV infection (Guma et al., 2004, 2006a,b; Della Chiesa et al., 2011). Similar interference with inhibitory receptor expression (e.g., NKG2A, KIRs) may be induced by acute infection, or exploited through KIR:HLA class I haplotype interaction, as for example is observed during infection with CMV (Muntasell et al., 2013), HCV (Khakoo et al., 2004; Knapp et al., 2010; Vidal-Castineira et al., 2010), or Chikungunya virus (Petitdemange et al., 2011).

Very little is known on NK cell phenotype and function during HTLV infection, in particular when HTLV-2 is considered.

Characterization of NK cell triggering and inhibitory receptors has been so far poorly addressed to understand the differences underlying HTLV-1 and HTLV-2 diseases.

Early reports on the definition of NK cell receptors point towards a decreased NK cell activity against HTLV-1-infected MT-2 cell lines (Fujihara et al., 1991; Zheng and Zucker-Franklin, 1992). Lysis of cell lines infected with HTLV-1 by *in vitro* activated NK cells was subsequently shown to occur and to depend on viral gene expression, that may be absent in some adult T cell leukemia (ATL) lines (Stewart et al., 1996). HTLV-1 antigen-driven proliferation may result in a ATL form, as shown in mice transgenic for Tax-1 (Grossman et al., 1995) and in humans with expansions in hypofunctional NK cells (Loughran et al., 1997). The proportion of circulating mature NK cells (CD56^+^ CD16^+^) is decreased in patients with TSP/HAM (Wu et al., 2000; Brito-Melo et al., 2002; Ndhlovu et al., 2009) and other innate immune cells (NKT) are decreased during TSP/HAM as well. In addition, more recent evidence suggests that NK cells may be targeted and induced to expand by HTLV-1 through a viral load-associated but Tax-independent mechanism (Norris et al., 2010).

Overall, there is need for further insight into NK cell phenotype and function during HTLV-1/-2 infection. More precise characterization of possible changes or modulation of triggering and inhibitory receptor expression on NK cells during chronic infection would help to better understand the mechanism(s) that are exploited by HTLV-1 to divert innate immune responses and downstream CD4^+^ and CD8^+^ T cell function. In particular, it is possible that different NK cell regulation induces clinical divergence spanning from the lack of symptoms to TSP/HAM and transformation to ATL, similar to what was observed for HCV infected patients clearing infection (Khakoo et al., 2004; Alter et al., 2011) or for HIV-infected patients who control virus replication (Elite Controllers) or without disease progression in LTNPs (Marras et al., 2013). In addition, in view of the compelling evidence of a remarkable NK cell activation during HIV-1 infection both in untreated patients (Fogli et al., 2004) and in successfully treated combined antiretroviral therapy (ART) patients (Lichtfuss et al., 2012) though belonging to AIDS or non-AIDS clinical groups (Bisio et al., 2013), understanding NK cell activation during HIV-/HTLV-1 co-infection needs improved focusing. These considerations should be extended to HTLV-2 infection *in vitro* and *in vivo*, since no data have been yet published on NK cells in mono-infected patients.

## ROLE OF MicroRNAs

MicroRNAs (miRNAs) are small single-strand non-coding RNAs that repress gene expression by inhibiting translation and inducing mRNA degradation (Ambros, 2004; Bartel, 2004). MicroRNAs can be encoded by both cellular and viral genomes (Berezikov et al., 2005; Grassmann and Jeang, 2008). Furthermore, viral miRNAs have been described not only for DNA viruses but also for RNA viruses as HIV-1 and BLV (Bennasser et al., 2004; Du and Zamore, 2007; Klase et al., 2013). MicroRNAs have been found to regulate up to 92% of the human genes (Miranda et al., 2006), and also to modulate viral gene expression. These alterations could be considered key mechanisms by which the virus imbalances immune system. In fact, as reported by several authors, immune response to invading agents as well as cellular proliferation and differentiation can be affected by host miRNAs (Chen et al., 2004; Fazi et al., 2005; Cobb et al., 2006; Li et al., 2007; Loffler et al., 2007; Ivanovska et al., 2008; Johnnidis et al., 2008; Carissimi et al., 2009; Faraoni et al., 2009; Huang et al., 2009; Lal et al., 2009; Curtale et al., 2010). The importance of RNA interference (RNAi) machinery in retroviral infection outcome was confirmed by recent findings that demonstrated how the miRNAs expression can be controlled by retroviruses (Sampey et al., 2012). Thus, the mechanism of RNAi mediated by miRNAs could be used by a virus to remain hidden from host immune surveillance by generating an advantageous cellular environments, and leading to adverse conditions for a co-invading agent.

MicroRNAs expression was studied during of HTLV-1 or HIV-1 infection but no data were so far reported for the HIV-1/HTLV-1 co-infection. Similarly, studies of miRNAs pattern during HTLV-2/HIV-1 co-infection have not been published up to date.

Concerning HIV-1, changes in host miRNA transcription levels have been detected in CD4^+^ purified cells from naive and LTNP HIV-1-mono-infected patients. The hypothesis that miRNAs either could directly influence viral RNA sequences, or could affect cellular factors involved in HIV replication were confirmed by several findings (Houzet and Jeang, 2011). More specifically, it was demonstrated that five cellular miRNAs recognize the 3′ end of HIV-1 mRNAs and are up-regulated in resting, but not activated, CD4^+^ T cells (Huang et al., 2007), providing evidence of a direct inhibition of HIV-1 replication by miRNAs. Two independent groups support this notion with the demonstration that HIV-1 *nef* gene contains a miR-29a targeted site that interferes with the replication of the virus (Ahluwalia et al., 2008; Nathans et al., 2009). Other authors found indirect inhibitory mechanisms miRNAs mediated. Specifically, it was found that miRNAs suppress viral gene expression by decreasing PCAF (P300/CBP-associated factor) expression and interfering with histone acetylation, and leading to HIV-1 latency (Triboulet et al., 2007). However, the deregulation of cellular miRNA expression which was shown to correlate to HIV-1 latency may also favor virus production (Han and Siliciano, 2007; Huang et al., 2007). Other miRNAs were involved in the different monocyte or macrophage susceptibility to the HIV infection (Wang et al., 2009, 2011).

Recently, it was shown that HIV-1 and other retroviruses as bovine leukemia virus can affect the expression of both host miRNAs and small virus-derived interfering RNAs (Klase et al., 2007, 2009; Houzet et al., 2008; Ouellet et al., 2008; Althaus et al., 2012; Kincaid et al., 2012; Schopman et al., 2012).

Similarly to HIV-1, it was demonstrated that HTLV-1 infection is responsible for alteration of host miRNAs profile (Bellon et al., 2009; Rahman et al., 2012). By profiling the expression of miRNAs, known to be involved in differentiation and proliferation of hematopoietic cells (Merkerova et al., 2008), abnormal levels of miR-223, miR-181a, miR-150, miR-142.3p, and miR-155, were detected in both primary ATL cells and in HTLV-1 cell lines (Bellon et al., 2009). Furthermore, these samples showed an altered expression of miR-155, miR-125a, miR-132, and miR-146 that play a role in the regulation of immune response (Bellon et al., 2009). A forced expression of miR-155, induced by Tax with the contribution of NF-κB and AP-1, has been found to enhance the proliferation of HTLV-1-infected cells (Tomita, 2012).

A loss of miR-31 was also observed in primary ATL cells, where the expression of this miRNA is epigenetically regulated, and was correlated with the constitutively activation of NF-κB which contributes to the oncogenic transformation (Yamagishi et al., 2012).

Concerning miRNAs biogenesis, low levels of Drosha enzyme, a key processor of miRNAs synthesis, were found in HTLV-1-infected cell lines and infected primary cells. In addition, *in vitro* studies revealed a nuclear co-localization of Tax and Drosha, and that the interaction between the two proteins leads to absence of cleavage of miRNAs by Drosha (Van Duyne et al., 2012).

MicroRNAs can target complementary sequences of both HTLV-1and HIV-1 transcripts and act as a major line of defense against retroviruses, but on the other hand viral proteins can interfere with RNAi machine directly or by altering the expression of cellular transcription factors. HTLV-1 Tax also interacts with CRE binding (CREB) protein, a key factor to viral transcription, in the presence of the HATs CREB binding protein (CBP), p300, and P/CAF to activate HTLV-1 gene expression while HTLV-2 Tax specifically cooperates with CBP and p300 but not with p300 associated factor to enhance transcription from the viral promoter (Tosi et al., 2006). Another mechanism by which HTLV-1 may influence the host cell miRNA expression profile is through the activation of host transcription factors. Important transcription factors and cellular kinases which interact directly with the viral protein Tax are CREB, serum-responsive factor (SRF), NF-κB, Cyclins D2 and D3, mitotic check point regulators (MAD1), cyclin dependent kinases (CDKs), the CDK inhibitors p16^INK4A^ and p21^(WAF1/CIP1)^, and the tumor suppressor p53 (Caron et al., 1993; Suzuki et al., 1994; Yin et al., 1995, 1998; Clemens et al., 1996; Colgin and Nyborg, 1998; Harrod et al., 1998; Gachon et al., 2000; Nicot et al., 2000; Xiao et al., 2001; Kashanchi and Brady, 2005; Easley et al., 2010). In particular, the NF-κB pathways activation is a hall mark of HTLV-1 infection and may be the result of direct interaction between Tax and the NF-κB regulatory subunit IKKγ (Sun and Yamaoka, 2005; Yasunaga and Matsuoka, 2011). Because numerous miRNA promoter sites are positively regulated by NF-κB, it can be inferred that the activation of NF-κB by Tax increases the expression of several host cell miRNAs (Li et al., 2012; Lukiw, 2012; Wang et al., 2012). One specific example is given by miR-155 which has been found to be up-regulated in HTLV-1-infected cells, as well as in a TNFα stimulated cell line through an NF-κB pathway (Bellon et al., 2009; Liu et al., 2011). Similar findings have already been described for HIV-1 Tat which activates NF-κB by acting through the same biochemical pathways used by a variety of other NF-κB inducers, reviewed in Baeuerle and Henkel (1994), and for other viral proteins, including Tax of HTLV-2, HBx, and MHBs of hepatitis B virus, and EBNA-2 and LMP of Epstein-Barr virus (Baeuerle and Henkel, 1994) and hemoagglutinin of influenza virus (Pahl and Baeuerle, 1995). Of particular interest is the role of miRNA in HTLV cellular transformation and recent findings demonstrate that alteration of the miRNA profile of infected cells leads to the development of ATL and HAM/TSP diseases (Sampey et al., 2012). In HIV-1 disease, a modification of chromatin by viral proteins and host cell miRNAs can contribute to the dysregulation of host cell miRNA expression and likely provides a key system used by the virus to modify host miRNA profiles (Bignami et al., 2012). Also, it was demonstrated that Tax-1 induces a prompt activation of chromatin remodeling factors as p300 and p/CAF (Rahman et al., 2012). The chromatin reorganization, affected by miRNAs which expression is in turns influenced by Tax proteins, drives the establishment of viral latent status (Aliya et al., 2012).

**FIGURE 1:**
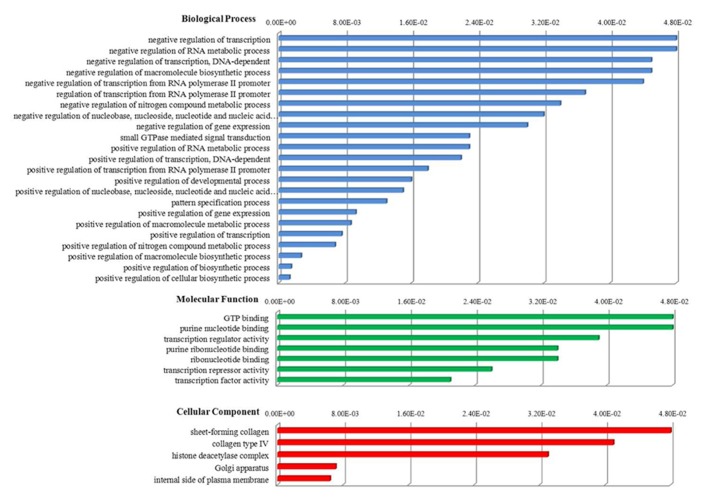
**Biological functions of host miRNAs belonging to HTLV-2 and HIV-1 retroviral signature.** One hundred and thirty-five predicted target genes were identified by miRDB database (http://mirdb.org). Functional annotations were provided by DAVID bioinformatics resources (http://david.abcc.ncifcrf.gov) on the basis of gene ontology (GO) resources that consider three different aspects of GO: biological process, cellular component, molecular function. The GO term selection was obtained using a significant *P*-value less than 0.05 as a statistical threshold. Functional annotations are sorted by score, from higher (minor significance) to lower (greater significance).

Recently, we studied miRNA profiles in CD4^+^ T-cells purified from HTLV-2-mono-infected patients and found evidence for a miRNA signature (miR-329, miR-337-5p, miR-379-5p, miR-503, miR-518d-3p, miR-203, miR-449a, miR-502-5p) that discriminates infected from uninfected subjects, similarly to HIV-1 (**Table 1**; Bignami et al., 2012). Furthermore, by analyzing in detail some functional aspects of the miRNAs belonging to retroviral signature we identified 135 predicted target genes that, on the basis of gene ontology (GO) resources, revealed the presence of three ontology aspects (**Figure 1**). Interestingly, we found that the most significant GO terms, i.e., positive regulation of macromolecule and cellular biosynthetic process, are related to the formation of substances carried out by individual cells.

**Table 1:** MicroRNAs equally expressed in HIV-1 and HTLV-2 vs HTLV-1 infection.

	HIV-1 vs healthy Bignami et al. (2012)	HTLV-1 vs healthy Pichler et al. (2008), Bellon et al. (2009), Ruggero et al. (2010), Rahman et al. (2012)	HTLV-2 vs healthy Bignami et al. (2012)
hsa-miRNA miR-329	Down	nd	Down
hsa-miR-337-5p	Down	nd	Down
hsa-miR-379-5p	Down	nd	Down
hsa-miR-503	Down	nd	Down
hsa-miR-518d-3p	Down	nd	Down
hsa-miR-203	Up	nd	Up
hsa-miR-449a	Up	nd	Up
hsa-miR-502-5p	Up	nd	Up

*nd indicates not determined.
*

With specific reference to the deregulated host miRNAs linked to the development of the HTLV-1 oncogenic or neurodegenerative diseases (**Table 2**), only miR-155, a key regulatory component of the innate immune response, is differentially expressed in HIV-1 and HTLV-2 infection. Thus, we speculated that a functional impact of cross-talk between miRNA pattern, and the subsequent multifunctional pathways, may occur during HTLVs/HIV-1 co-infection.

**Table 2:** MicroRNAs either up- or down-regulated in HTLV-1 infected cells vs HIV-1 and HTLV-2 infection.

	HIV-1 vs healthy Bignami et al. (2012)	HTLV-1 vs healthy Pichler et al. (2008), Bellon et al. (2009), Ruggero et al. (2010), Rahman et al. (2012)	HTLV-2 vs healthy Bignami et al. (2012)
hsa-miRNA miR-21	ns	Up	ns
hsa-miR-24	nd	Up	ns
hsa-miR-93	nd	Up	ns
hsa-miR-132	nd	Down	ns
hsa-miR-143-3p	nd	Up	nd
hsa-miR-146a	nd	Up	ns
hsa-miR-149	ns	Down	ns
hsa-miR-155	ns	Up	Up
hsa-miR-223	ns	Up	ns
hsa-miR-873	Down	Down	ns
hsa-miR-150	nd	Up	ns
hsa-miR-142-5p	nd	Up	ns
hsa-miR-181a	nd	Down	ns
hsa-miR-125a	Up	Down	Up
hsa-miR-146b	ns	Down	ns

*ns indicates not significant; and nd not determined.
*

The assessment of how the altered profiles of miRNA expression can influence viral replication and latency, as well as the efficiency of host defenses, may be useful for understanding the basis of the retroviral related modifications of cellular pathobiology and immunologic control.

## CONCLUSION

Is the retroviral interference relevant to HIV-1 infection? In this review we discussed how HIV-1/HTLVs co-infection can either positively or negatively affect the course of HIV-1 disease. In particular, co-infection with HTLV-2 seems to confer immunological benefits in patients with HIV-1. By contrast, HTLV-1 is mainly associated to HIV-1 disease progression and to an increased risk of TSP/HAM and ATL.

An overall picture of different effects of HTLVs on HIV infection is emerging. The widely diverging effect of HTLV-1 and HTLV-2 on the clinical course of HIV-1 progression is remarkable, and the findings initially reported for HTLV-2 are particularly surprising. The possibility that specific co-infections may improve the clinical course of HIV-1 infection by directly or indirectly interfering with HIV-1 cell entry, replication and spread, originally proposed by the study of HIV-1/HTLV-2-infected cells (Casoli et al., 2000) has been confirmed by subsequent work on GB virus C which is able to infect B cells and CD4^+^ or CD8^+^ T lymphocytes (George et al., 2006).

Modulation of the cytokine/chemokine network represents a major element of shift dynamics that regulates the co-existence of several infections. Thus, cytokines and chemokines might be considered strategic weapons in the bid to gain benefits to the infecting agents. Since a poor Th1 response and a dominant Th2 response have been implicated in the pathogenesis and progression of HIV infection (Clerici and Shearer, 1993, 1994), HTLV-2 priming for a Th1 response via up-regulation of IFN-γ expression may contribute to the “protective” effect of HTLV-2 infection on HIV-1 disease progression. In the case of HTLV-1 co-infection, high frequencies of activated HTLV-1-infected CD4^+^ T cells can give a boost to HIV-1 replication.

An enhanced secretion of CC-chemokines, in particular of CCL3L1, was ascribed to the transactivating function of Tax-2 and the original studies of HTLV-2/HIV-1 co-infection proposed this as a key mechanism of retroviral interference. The CCL3L1 isoform down-regulates CCR5 co-receptor for HIV-1 entry leading to a LTNP status in co-infected individuals with high HTLV-2 proviral load (Pilotti et al., 2007).

In the same manner, GBV-C acquisition via blood transfusion increases the secretion of CCL5, CCL3, CCL4, and CXCL12 and by means of their NS5A and E2 proteins support the deregulation of co-receptors, thus inhibiting HIV entry and resulting in a reduced mortality in patients with advanced HIV-1 disease (Tillmann et al., 2001; Bhattarai and Stapleton, 2012). Similarly to what occur in HIV-1/HTLV-1 co-infection, HIV-1 disease progression is faster in individuals affected by HCV or HSV co-infection (Van Asten and Prins, 2004; Corey, 2007).

Analysis of JAK/STAT regulation during HTLV-2 infection provides some clues of intervention to interfere with HIV-1 replication by taking advantage of pathway interference instead of enzymatic inhibition of viral enzymes. The lack of knowledge of HTLV-1 mediated activation pathway interference has probably limited efforts in this direction.

Concerning innate immune responses in HIV-1 co-infection with either HTLV-1 or HTLV-2, no data are so far available for NK cell function, and little is known on other innate immune cellular mechanisms. The observed effect of proviral load, but not of Tax-1, on NK cell proliferation during HTLV-1 infection (Norris et al., 2010), is likely to impact also on HIV-1/HTLV-1 co-infected patients, leading to enhanced NK cell activation and possibly disease progression. On the contrary, when considering HTLV-2/HIV-1 co-infection, evidence of HTLV-2 interference with STAT/JAK pathways could possibly be linked to a decreased HIV-1-associated NK cell activation. Further work in this direction is needed to improve our understanding of the mechanism(s) associated with the positive effect of HTLV-2 on HIV-1 disease course, and of the underlying causes leading to AIDS progression by HTLV-1/HIV-1 co-infection and/or to HTLV-1 associated morbidity.

Since miRNAs have been correlated with viral life cycle, they represent good candidates among the top cellular factors to be used by HTLVs to favor their own replication. In the case of co-infection with HIV-1, HTLV proteins were found to interact with cellular chromatin modifying enzymes and with cellular transcription and other immune factors. The interaction of HTLV Tax proteins with cellular factors results in the alteration of miRNAs profile that in turns can activate transcription and consequently viral replication. Furthermore a likely interplay between two competing mechanisms is taking place: the ability of Tax to manipulate chromatin structure and the innate host cellular defense mechanism of RNAi to regulate pathogen gene expression.

HTLV/HIV-1 co-infection can be considered as a useful model for the study of new strategic approaches for HIV-1 vaccine development, as suggested by the finding that the exposure of HTLV-2 infected macaques to SIV_mac251_ was not accompanied by an exacerbation of SIV_mac251_ infection (Gordon et al., 2010).

## Conflict of Interest Statement

The authors declare that the research was conducted in the absence of any commercial or financial relationships that could be construed as a potential conflict of interest.
